# Mitochondria-Targeted Nanomedicine for Enhanced Efficacy of Cancer Therapy

**DOI:** 10.3389/fbioe.2021.720508

**Published:** 2021-08-19

**Authors:** Yan Gao, Haibei Tong, Jialiang Li, Jiachen Li, Di Huang, Jisen Shi, Bing Xia

**Affiliations:** ^1^ College of Science, Key Laboratory of Forest Genetics and Biotechnology (Ministry of Education of China), Nanjing Forestry University, Nanjing, China; ^2^ Drug Research Program, Division of Pharmaceutical Chemistry and Technology, University of Helsinki, Helsinki, Finland

**Keywords:** mitochondria targeting, nanomedicine, enhanced efficacy, photothermal therapy (PTT), chemoteraphy, immunotherapy

## Abstract

Nanomedicines have been designed and developed to deliver anticancer drugs or exert anticancer therapy more selectively to tumor sites. Recent investigations have gone beyond delivering drugs to tumor tissues or cells, but to intracellular compartments for amplifying therapy efficacy. Mitochondria are attractive targets for cancer treatment due to their important functions for cells and close relationships to tumor occurrence and metastasis. Accordingly, multifunctional nanoplatforms have been constructed for cancer therapy with the modification of a variety of mitochondriotropic ligands, to trigger the mitochondria-mediated apoptosis of tumor cells. On this basis, various cancer therapeutic modalities based on mitochondria-targeted nanomedicines are developed by strategies of damaging mitochondria DNA (mtDNA), increasing reactive oxygen species (ROS), disturbing respiratory chain and redox balance. Herein, in this review, we highlight mitochondria-targeted cancer therapies enabled by nanoplatforms including chemotherapy, photothermal therapy (PTT), photodynamic therapy (PDT), chemodynamic therapy (CDT), sonodynamic therapy (SDT), radiodynamic therapy (RDT) and combined immunotherapy, and discussed the ongoing challenges.

## Introduction

Cancer still presents a major threat to human health, causing over eight million related death every year and enormous economic loss (Ferlay et al., 2015). Conventional clinical cancer treatments are majorly radiotherapy (RT), surgery and chemotherapy. RT employs radiation to kill local tumor tissues. Surgery involves directly removal of tumor entity or whole tissue. And chemotherapy interferes with the cell division of rapidly dividing cells non-specifically through, for example, inhibiting DNA replication, or separating newly formed chromosomes. These conventional cancer treatments have their limitations or drawbacks in practical clinical applications. Surgery treatment is difficult to avoid tumor residuals, while both RT and chemotherapy lead to systematic toxicity, causing different degrees of side effects to normal tissues, which limit their clinical applications. Therefore, accurate and precise targeting treatments of tumor cells has drawn increasing research interest in recent years to improve therapeutic efficacy and reduce undesirable harms to normal tissues.

Nanoparticles provide powerful tools for targeting therapies to tumor tissues for improved anticancer effects (Hrkach and Langer, 2020). Various nanocarriers have been designed and developed to deliver anticancer drugs more selectively to tumors than to healthy tissues. Anticancer drugs can be delivered by nanocarriers into the tumors by either passive or active mechanisms. The passive accumulation in tumor is majorly based on enhanced permeation and retention (EPR) effect of nanoparticles (Fang et al., 2011). EPR effect results from enlarged blood capillary vessels of tumors in comparison to normal tissues, so nanoparticles in the size range from 50 to 500 nm efficiently enter the tumor intersitium from blood vessels. In addition to the passive accumulation, nanoparticles with active targeting performance are also employed to deliver drugs, by modifying specific ligands with high affinity to cancer cells (*e.g.* polypeptides (Fang et al., 2020; Kang et al., 2020), proteins, antibodies (Chen et al., 2012; Pan et al., 2021), folic acids (Mitra et al., 2018) and aptamers (Tang et al., 2020; Tian et al., 2021)). On this basis, the unique advantages of nanocarriers have been widely demonstrated including long-term sustained (Xia et al., 2019) and controlled release (Xia et al., 2014) and the integration with more diagnostic or therapeutic modality such as multimodal imaging (Gao et al., 2017; Xia et al., 2017; Liu et al., 2019), photodynamic therapy (PDT) (Lucky et al., 2015; Wen et al., 2020), photothermal therapy (PTT) (Xia et al., 2016; Yang et al., 2017) and immunotherapy (Meraz et al., 2012; Secret et al., 2013). However, the major limitation of nanomedicine lies in the insufficient anticancer therapeutic efficacy, thus there is an urgent demand to further improve their therapy efficacy. In this sense, more and more investigations are not only limited in the delivery of anticancer drugs and focusing therapy by nanocarriers to tumor tissues and cancer cells, but also take a step further to deliver drugs or therapy to specific subcellular organelles, in order to increase efficacy, circumvent drug resistance, etc. The targeting subcellular organelles include lysosomes (Jeanbart and Swartz, 2015), mitochondria (Murphy and Smith, 2000), endoplasmic reticulum, Golgi apparatus and nucleus (Harisa and Faris, 2019).

Mitochondria are subcellular organelles of vital importance for eukaryotic cells for many reasons. Firstly, they are considered as “energy factory” for cells with the generation of adenosine triphosphate (ATP) to maintain life activities. Mitochondria provide the sites of oxidative metabolism for eukaryotes and ATP are produced as a result of this oxidation process. Secondly, mitochondria are the main places where cells undergo aerobic respiration to regulate cellular ROS and redox balance. In the process of oxidative phosphorylation, a small number of electrons will reduce oxygen in advance and form ROS such as superoxide. Thirdly, they are key regulators of cell intrinsic apoptosis mediated by caspase-9/caspase-3 pathway, thus participate in many important cellular events including cell differentiation, proliferation, apoptosis and necrosis. Finally, mitochondria are also crucial to the regulation of intracellular calcium homeostasis. Mitochondria store calcium ions and can act synergistically with structures such as the endoplasmic reticulum and extracellular matrix, to control the dynamic balance of calcium ion concentration within a cell. They are also involved in calcium signaling during apoptosis. Accordingly, mitochondria play an important role on the regulation of cancer occurrence, metastasis, or recurrence (Ishikawa et al., 2008; Porporato et al., 2014; Pustylnikov et al., 2018). In one hand, fast proliferation of cancer cells requires more energy than normal cells, which supposed to be supplied by mitochondria. In the otherhand, cancer cells are commonly dysregulated in apoptotic machinery and resistant to mitochondria-mediated apoptosis. Mitochondria-targeted cancer therapy intends to induce cancer cell intrinsic apoptosis by disturbing the respiratory chain and redox homeostasis, increasing ROS levels, and damaging mitochondria DNA (mtDNA). Disturbing the mitochondria respiratory chain reduces ATP production and increases endogenous ROS generation. Excessive ROS, either endogenous or exogenous, not only damages mtDNA, but also results in the entering of calcium ions into the mitochondria matrix, followed by osmotic swelling of the mitochondria and outer mitochondria membrane rupture. mtDNA encode 13 polypeptides of mitochondria and all these 13 polypeptides participate in the respiratory chain (Wallace, 1995; Saraste, 1999). Thus, damaging mtDNA would also dysfunction the respiratory chain. As a whole, all the above-mentioned disturbance to mitochondria would interfere their functions and trigger cellular intrinsic apoptosis. Compared to non-targeting treatment, more precise mitochondria-targeted treatment exerts enhanced anticancer efficacy.

**FIGURE 1:**
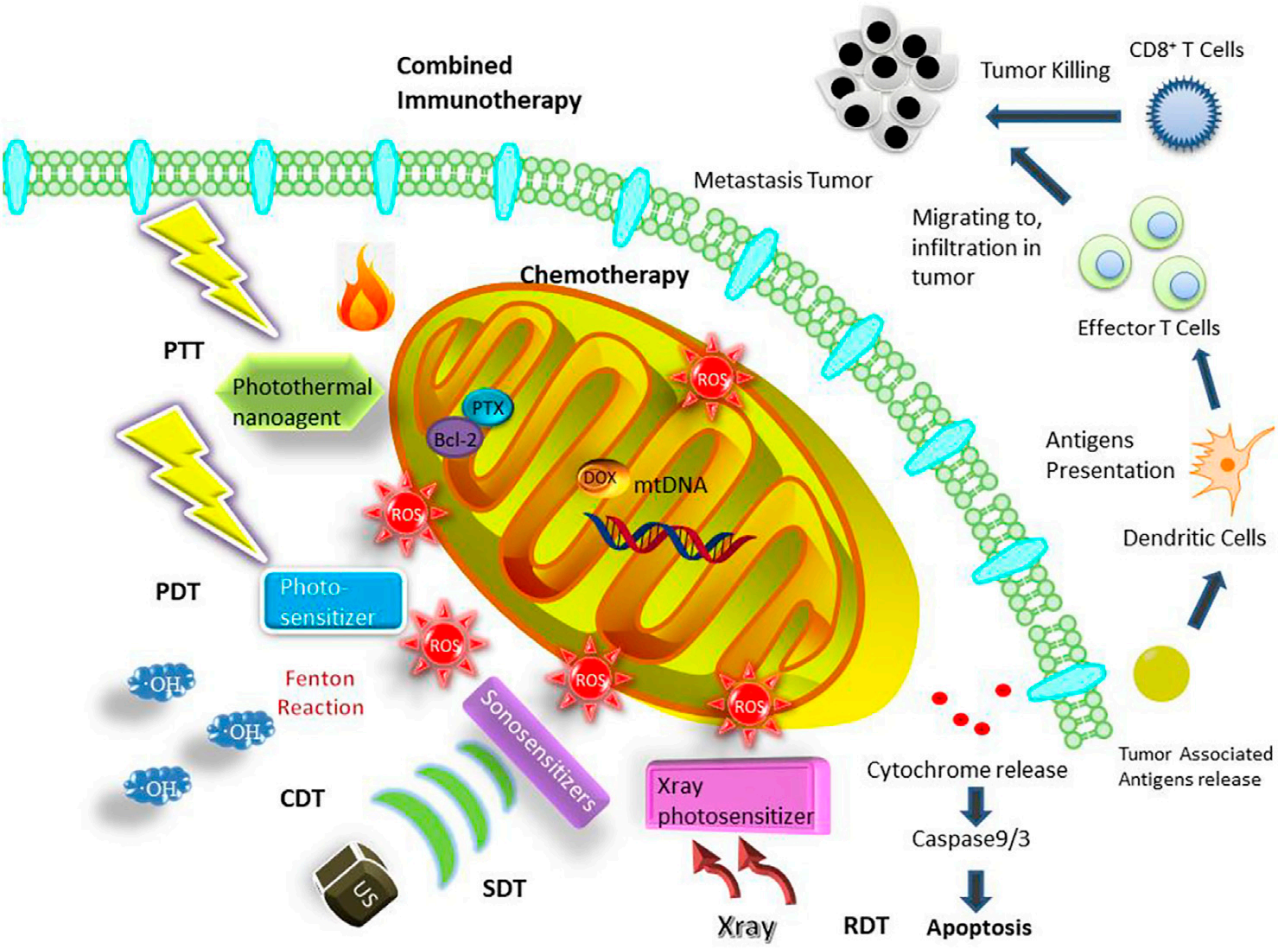
Mitochondria-targeted cancer therapies enabling by nanoplatforms including chemotherapy, PTT, PDT, CDT, SDT, RDT and combined immunotherapy.

The outer mitochondrial membrane (OMM) has no membrane potential; however, the inner mitochondrial membrane (IMM) folded with large surface area containing a variety of proteins involved in oxidative respiration chain has a membrane potential of approximately −180 mV, resulting in their high transmembrane potential. Considering that molecules should be lipophilic and cationic to possess high affinity to mitochondria, typical mitochondriotropic ligands are mostly delocalized lipophilic cations (DLCs), including triphenylphosphonium (TPP) (Han et al., 2014), dequalinium (DQA) (Weiss et al., 1987; Mallick et al., 2019), rhodamine 123 (Jeannot et al., 1997), Cy5.5 (Jiang et al., 2019) and some transition metal complexes (Yi et al., 2019) (Table 1). In addition, mitochondriotropic supramolecules have mitochondria-penetrating peptides (MPP) (Chamberlain et al., 2013), Szeto-Schiller (SS) peptides (Zhao et al., 2004), liposomes (Yasuzaki et al., 2010). For example, HIV-1 encoded trans-activator protein was the originally founded cell penetrating peptide (Green and Loewenstein, 1988), with guanidine and amino group on arginine and lysine respectively rendering the strong positive charge. Arginine and lysine are retained in MPP for providing positive charge while phenylalanine and cyclohexylalanine are added to provide lipophilic moieties (Horton et al., 2008). SS peptide is an aromatic cationic peptide with alternating aromatic ring and amino acid residues. The guanidine group of arginine in SS peptide provides cations while the aromatic group is lipophilic (Szeto, 2006). Liposomes are easily fused into mitochondria because the mitochondrial membrane is also phospholipid bilayer. Generally, the fabrication of mitochondria-targeting nanomedicine is designed by incorporating these cationic and lipophilic onto the nanocarriers. After modified with mitochondria-targeting ligands, multifunctional nanoplatforms have been constructed for cancer therapy including chemotherapy, PTT, PDT, chemodynamic therapy (CDT), sonodynamic therapy (SDT), radiodynamic therapy (RDT) and combined immunotherapy, which are summarized (Figure 1) in this review. Although a lot of works on mitochondria-targeted drug delivery have been summarized (Hou et al., 2018; Murphy and Hartley, 2018; Zhou et al., 2019; Liew et al., 2021), the reviews on mitochondria-targeted nanotechnology for cancer treatment are few (Qin et al., 2021), thus our report mainly focus on this view point.

**TABLE 1:** A summarization of mitochondria-targeted cancer therapies in which nanoparticles used, with their targeting ligands and tumor cells are used to demonstrate their efficacies.

Nanoparticles	Targeting ligands	Size	Therapy	Test objects	Reference
TPP-PEG-liposome-paclitaxel (TPP-PEG-L-PTX)	TPP	159.8 ± 39.0 nm by DLS	chemotherapy	HeLa, 4T1 cells and 4T1 tumor model	Biswas et al. (2012)
Multi-walled carbon nanotubes-Rhodamine110 (MWCNTs-Rho)	rhodamine-110	30–60 nm by TEM	chemotherapy	A2780 cells	Yoong et al. (2014)
TPP-Pluronic F127-hyaluronic acid (TPH/PTX)	TPP	142 ± 8.35 nm by DLS	chemotherapy	A549/ADR cells and tumor model	Wang et al. (2020)
TPP-coumarin iron oxide (Mito-CIO)	TPP	15 nm by TEM	PTT	HeLa cells and A549 tumor model	Jung et al. (2015)
Supra-carbon dots-MPP (SCDs-MPP)	Mitochondria-targeting peptide	20 nm by TEM	PTT	HepG2 cells	Shen et al. (2020a)
Rhodamine-110-Bovine serum albumin@ CuS (R-BSA@CuS)	rhodamine-110	15.9 nm by DLS	PTT	MCF-7 cells and 3D multicellular MCF-7 tumor spheroids	Tong et al. (2021)
Human serum albumin- poly(ethylene oxide)-TPP- Ruthenium complexes (cHSA-PEO-TPP-Ru)	TPP	40 nm by DLS	PDT	OCI-AML3 cells	Chakrabortty et al. (2017)
poly(ethylene glycol)-b-poly(2-(isopropylamino) ethyl methacrylate) (mPEG-b-PDPA)-Cy5.5-TPP-pyropheophorbide-a (M-TPPa)	TPP	24.33 ± 1.86 nm by DLS	PDT	HO8910 cells and tumor model	Qi et al. (2019)
2,5-bis(6-bromohexyl)-3,6-bis(5-(4-(diphenylamino)phenyl)thiophen-2-yl)-2,5-dihydropyrrolo[3,4-c]pyrrole-1,4-dione (DPP)/DPP+/DPP 2+	Imidazole	200, 240, 260 nm by DLS	PDT PTT	HeLa cells and mouse cervical U14 tumor model	Li et al. (2020b)
poly(ε-caprolactone)-poly(ethylene glycol) liposomes-IR780-metformin (PEG-PCL-IR780-MET)	IR780	60.90 nm by DLS	PDT PTT	MKN-45P cells and tumor model	Yang et al. (2020b)
photothermal bacterium-Zeolitic imidazole framework-90- methylene blue (PTB@ZIF-90/MB)	ZIF-90	N/A	PDT PTT	Mouse colon cancer (CT26) cells and tumor model	Chen et al. (2020)
cyclometalated iridium(Ⅲ) complex- MnFe_2_O_4_ (Ir@MnFe_2_O_4_)	cyclometalated iridium(Ⅲ) complex	11.24 nm by TEM	MHT CDT	HeLa cells and tumor model	Shen et al. (2020b)
Au-TiO_2_-AS1411 aptamer- TPP (Au-TiO_2_-A-TPP)	TPP	68.1 nm by DLS	SDT	MCF-7, A549, NHDF cells and MCF-7 tumor model	Cao et al. (2019)
Ce6-DOX-Hollow polydopamine-TPP (CDP@HP-T)	TPP	∼250 nm by TEM	SDT chemotherapy	4T1 cells and tumor model	An et al. (2020)
Hf-Bis (2,2’-bipyridine) (5,5’-di(4-benzoato)-2,2’-bipyridine) ruthenium (II) chloride-Ru(bpy)_3_^2+^ (Hf-DBB-Ru)	Ru(bpy)_3_^2+^	98.1 ± 4.1 nm by DLS	RDT	Murine colon adenocarcinoma (MC38) cells and tumor model	Ni et al. (2018)
catalase@silica-Ce6-CTPP-dimethylmaleic anhydride (DMMA)-PEG (CAT@S/Ce6-CTPP/DPEG)	(3-carboxypropyl) triphenylphosphonium bromide (CTPP)	∼100 nm by TEM	PDT combined immunotherapy	4T1 and tumor model	Yang et al. (2018)
HA-PEI-iRGD@modified Ce6 (TPP-PEI-Ce6) + Cas9-Ptpn2 (HPR@CCP)	TPP	115 ± 5.8 nm by DLS	PDT combined immunotherapy	B16F10 cells and tumor model	Yang et al. (2020)

## Mitochondria-targeted Nanomedicine for Enhanced Chemotherapy

Chemotherapy directed to mitochondria of cancer cells may bring about enhanced cytotoxicity, majorly through disturbing the functions of mitochondria and triggering mitochondria-mediated apoptosis. Firstly, this enhanced therapeutic efficacies generally result from drugs that solely or partially act on mitochondria. For the latter case, Paclitaxel (PTX) was originally believed to bind to microtubules in the cytoplasm, stabilizing microtubules to inhibit mitosis and proliferation of cancer cells. However, recently PTX was also found to bind to anti-apoptotic protein Bcl-2 family in mitochondria and open the mitochondrial permeability transition pore (MPTP) channels (Ferlini et al., 2009), which control the mitochondrial permeability of small molecules, then depolarize mitochondria membrane, facilitating the initiation of apoptosis. Therefore, the underlying mechanism of mito-toxicity provides possibilities of targeting PTX to mitochondria for augmented efficacy. In the work reported by Biswas et al. (2012), the delivery of PTX based on liposomes modified with TPP-polyethylene glycol-phosphatidylethanolamine (TPP-PEG-PE) to mitochondria targets exert enhanced antitumor effects. Here, TPP was employed as mitochondria-targeting ligand while PEG-PE increased the biosafety of the nanocarrier. TPP-PEG-PE modified liposomes (TPP-PEG-L) were less cytotoxic compared to stearyl TPP modified liposomes (STPP-L) or PEGylated STPP-L. PTX delivered by TPP-PEG-L showed amplified PTX related cytotoxicity compared to PTX delivered by unmodified plain liposomes (PL) when co-incubated with both human cervical cancer (HeLa) cells and murine breast cancer cells (4T1) cells at 650 ng ml^−1^ for 24 or 48 h. This augment in anticancer efficacy was also demonstrated *in vivo*, ascribed to mito-toxicity of PTX.

Secondly, many model drugs act by impairing DNA, and when they are directed to mitochondria, they would also damage mtDNA (Chamberlain et al., 2013). However, unlike intracellular nuclei, multiple DNA repair systems are known to reverse the detrimental effects of drugs, such repair mechanisms are less efficient in the mitochondria (Han et al., 2014). Lack of protective histones renders mtDNA much more vulnerable than nuclear DNA (Preston et al., 2001). Therefore, chemotherapy directed to mitochondria may lead to augmented efficacy than non-targeting ones. Yoong and coworkers (2014) functionalized multi-walled carbon nanotubes (MWCNTs) with rhodamine-110 (Rho) as a vehicle to deliver platinum (Ⅳ) prodrug of cisplatin (PtBz) to mitochondria for the disruption of mtDNA. Here, Rhodamine-110 was chosen as mitochondria-targeting ligand, and its native fluorescence is an advantage that enables the tracking of nanocarriers via fluorescent imaging. Compared to its non-mitochondrial targeting counterpart of MWCNT conjugated with fluorescein (MWCNT-Fluo), MWCNT-Rho (PtBz) was seven to eight times potent in drug activity than MWCNT-Fluo (PtBz). While the PtBz loading efficiencies are similar between these two delivery systems, this enhancement was assumed to be attributed to selective delivery to mitochondria leading to mitochondrial DNA impairment. Synergistic effect was also tested by co-loading MWCNT-Rho nanoncarrier with PtBz and chemo-potentiator, 3-bromopyruvate (BP). BP inhibits hexokinase Ⅱ-voltage dependent anion channel (VDAC) interaction at mitochondrial outer membrane, which would disrupt cell metabolism and make them more susceptible to apoptosis. MWCNT-Rho (PtBz + BP), compared to free PtBz, PtBz + BP and MWCNT-Rho (PtBz), has amplified cytotoxicity in a variety of cancer cell lines, demonstrating the synergistic effect combing BP as a chemo-potentiator. In addition, the mechanism was further explored, revealing that MWCNT-Rho (PtBz + BP) resulted in two times decrease in mitochondrial membrane potential (MMP) than free PtBz in human ovarian and breast cancer cell lines, measured by JC-1 probe, indicating additional mitochondrial injury. JC-1 probe, 5,5’,6,6’-tetrachloro-1,1’,3,3’-tetraethylbenzimidazolcarbocyanine iodide is used as an indicator to detect the depolarization of MMP. JC-1 has two states: a monomer and an aggregate. In normal cells, the MMP has polarity and JC-1 rapidly enters the mitochondria through the polarity of the mitochondrial membrane and forms an aggregate state that emits a red light with the increase of concentration. When cells undergo apoptosis, the MMP becomes depolarized, JC-1 is released from the mitochondria, the concentration decreases and reverses to the monomer state that emits green fluorescence and the red light weakens.

**FIGURE 2:**
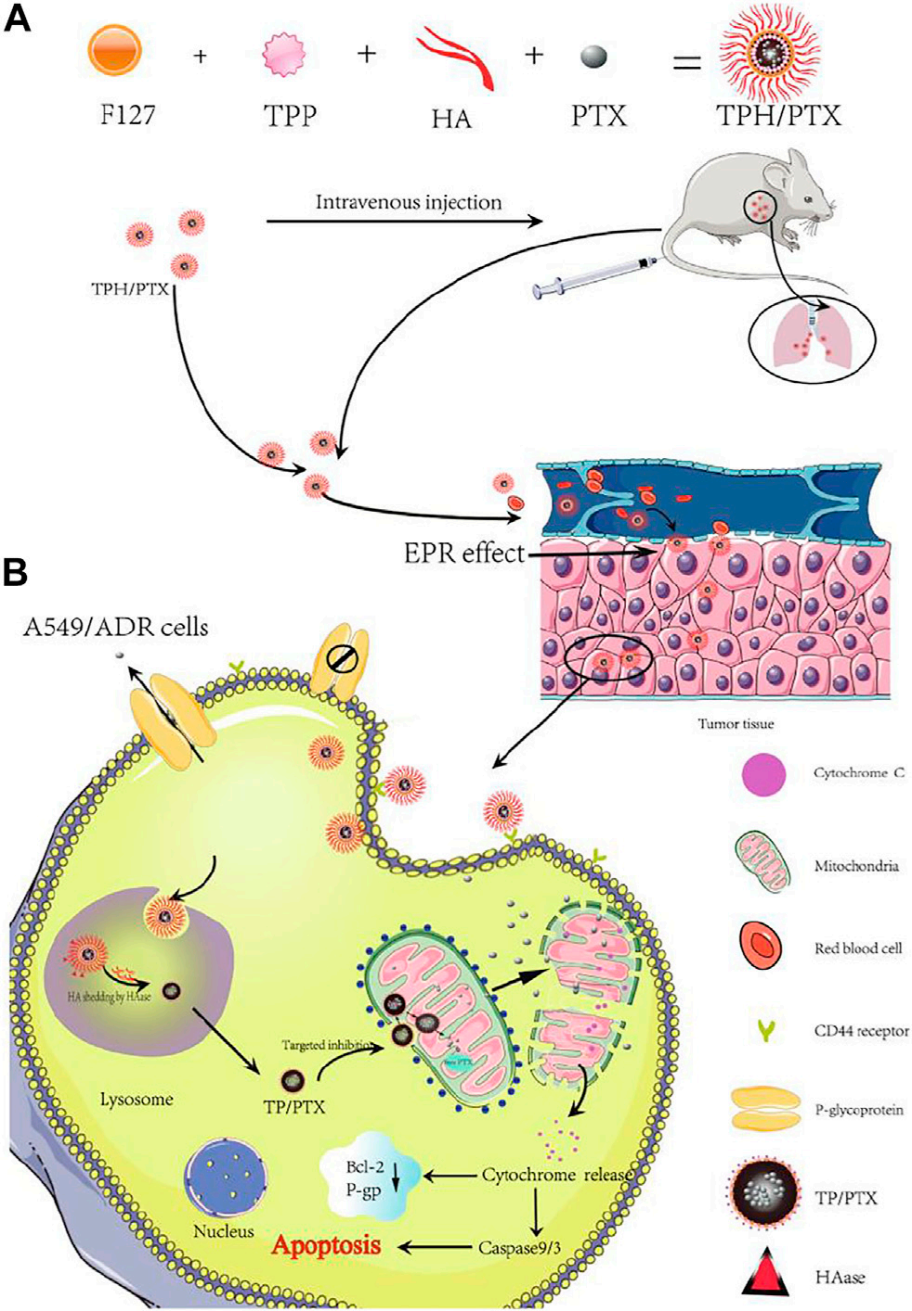
Schematic representation of the assembly of TPH/PTX nanomicelles and the transporting pathway *in vivo* of TPH/PTX nanomicelles. Reproduced with permission from Wang et al. (2020). Copyright (2020) BMC.

Thirdly, directing standard drug to mitochondria was also considered an approach to overcome multidrug resistance (MDR) of cancer cells. MDR often results from drug efflux pump of cancer cells, and dramatically compromises the effectiveness of drugs. In addition, targeting model drug with mito-toxicity to mitochondria would injure mitochondria and cut off ATP supply for the overexpression of drug efflux pump to reverse the drug resistance. In order to reduce drug resistance of lung cancer, Wang and co-workers (2020) developed a nanocarrier composed of TPP, Pluronic F127 and hyaluronic acid (HA) to deliver model drug PTX to mitochondria to restrain ATP production, thus reduce the expression of drug efflux pump. In drug resistant cancer cell lines, the efficacy of PTX was greatly limited by drug efflux pump. When PTX loaded into nanocarrier forming TPP-Pluronic F127-HA/PTX (TPH/PTX) nanomicelles directed to mitochondria (Figure 2), for lung cancer cell line A549, the apoptosis percentage after treating with Taxol (the pharmaceutical formulation of PTX), TP/PTX and TPH/PTX were 43.1, 29.1 and 48.4%. Taxol itself, not delivered by TPH nanomicelles, can also cause apoptosis. Whereas for drug resistant lung cancer cell line A549/ADR, the rate of apoptosis induced by Taxol alone was only 2.15%. PTX delivered by TPH greatly increased the apoptosis rate of A549/ADR cell to 22.10%. Mitochondrial outer membrane permeabilization proves that the cytotoxicity mechanism was attributed to mitochondrial apoptosis pathway triggered by PTX. In an A549/ADR xenograft tumor model and a drug-resistant breast cancer bearing mouse model with lung metastasis, it is also shown that compared to Taxol alone, or TP/PTX, TPH/PTX exhibited significantly improved anticancer efficacy. These results together proved that PTX delivered by mitochondria-targeting TPH results in mito-toxic effects and alleviates drug resistance.

In conclusion, chemotherapy targeted to mitochondria delivered by nanoplatform brings about enhanced efficacy by initiating mitochondria mediated apoptosis *via* a variety of mechanisms including opening up MPTP channels or disturbing mtDNA, and overcome drug resistance by blocking the energy supply for drug resistant proteins.

## Mitochondria-targeted Nanomedicine for Enhanced Phototherapy

### Mitochondria-targeted Nanomedicine for PTT

The principle of hyperthermia therapy is employing physical energy to raise temperature of the tumor tissues, inducing the apoptosis or necrosis of cancer cells. Recently, PTT has drawn increasing interest due to its reduced invasiveness and less harm to nearby normal tissues as it only involves local temperature elevation. In PTT, heat is produced by photothermal nanoagents converting light energy to heat upon near-infrared (NIR) laser irradiation, resulting in localized cancer cell death and eventually thermal ablation of tumors. The first generation of these photothermal nanoagents are noble metal nanoparticle, including Au (Cheng et al., 2009), Ag (Bian et al., 2018) and Pt (Tang et al., 2018). The second generation are carbon-based materials like graphene (Chen et al., 2016), carbon nanotubes (Feng et al., 2014) and other carbon based nanomaterials (Liu and Liang, 2012; Tu et al., 2014). The third generation are metal chalcogenide compounds, like CuS (Xiao et al., 2020) and ZnS (Wu et al., 2016). And the latest category is organic reagents, like IR780 (Sheng et al., 2014) and Prussian blue (Zhou et al., 2018). Compared to conventional nanoplatform based PTT, photothermal nanoagents are designed to specifically accumulate in mitochondria, resulting in dramatic temperature rise focused in mitochondria area for a given amount of heat, a direct initiation of mitochondria-mediated apoptosis or necrosis, and further an enhanced photothermal damage to cancer cells (Slimen et al., 2014; Ahmed et al., 2015). This is because mitochondria are particularly sensitive to heat stress caused by hyperthermia, which can heavily disturb their functions or directly destruct them.

In order to augment the hyperthermic cytotoxicity and direct generated heat to mitochondria, Jung and coworkers (2015) designed a mitochondria-targeting photothermal iron oxide NPs by modifying TPP. For the purposes of fluorescent labeling, the iron oxide NP was also conjugated with coumarin to form mitochondrial-targeted coumarin iron oxide (Mito-CIO) nanoparticle. The photothermal conversion of Mito-CIO was rather efficient as temperature of Mito-CIO solution of 7.0 mg ml^−1^ increased by 13°C upon irradiation with 740 nm at 2.0 W cm^−2^ laser for only 2 min. The temperature raises in cells after uptake the nanomaterial was also distinct. After cells were treated with Mito-CIO or coumarin iron oxide (CIO), Mito-CIO induced more cell mortality (89.90%) than CIO (42.58%) after 20 min 0.04 W cm^−2^ NIR irradiation, while their intracellular concentration was almost identical. Furthermore, co-localization experiment with organelle trackers showed that Mito-CIO and CIO were mainly localized within mitochondria and endoplasmic reticulum respectively. These results indicated that TPP as a vector to direct Mito-CIO to mitochondria resulted in hyperthermia in mitochondria, which is mainly responsible for substantial improvement of the cytotoxicity on cancer cell. Annexin V-FITC and PI revealed that the cytotoxicity was attributed to a combination of cell apoptosis and necrosis. Moreover, the therapeutic effects of Mito-CIO were also evaluated in A549 cell xenografted mice model after intravenous injection of materials. Mito-CIO group showed strongest tumor suppression *in vivo* after six doses with NIR irradiation when compared to IO and CIO, proving enhanced mitochondria-directed photothermal therapeutic efficacy of Mito-CIO.

In another work, novel supra-carbon dots (SCDs) were conjugated with mitochondria-targeting peptide to amplify the photothermal therapeutic efficacy for cancer cells (Shen J. et al., 2020). Carbon dots (CDs) are widely used in biological applications because their excellent biocompatibility. However low visible-NIR adsorption limits their applications in tumor imaging or cancer phototherapy. By controlling the pH during synthesis of CDs, SCDs with high visible-NIR adsorption were developed. Mitochondria-targeting peptide (MLS, MLALLGWWWFFSRKKC) and the cancer membrane penetrating peptide (RGD, CGPDGRDGRDGRDGR) were conjugated on SCDs to fabricate SCDs-MT with cancer cell selectivity and mitochondria-targeting ability. When irradiated with 4.0 W cm^−2^ 808 nm laser for 10 min, the temperature elevation of the as-prepared SCDs was 5.5 times higher than that of the CDs, indicating their high photothermal conversion efficiency. And the photothermal conversion ability of the SCDs could still be maintained under either acid or alkaline conditions. When pH changes from acid to alkaline, the intensity of maximum adsorption of SCDs increased by 47%. This is favorable for their use in mitochondrial PTT as the mitochondria pH environment is 8.0. SCDs-MT was applied for damaging cancer cells by PTT. After exposed to laser irradiation for 10 min, the viability difference between liver cancer cells (HepG2) and normal liver cells reached 70%, demonstrating SCDs-MT has high selectivity and specificity to cancer cells. For HepG2 treated with 500 μg ml^−1^ SCDs-MT, 10 min laser irradiation induced about 60% death while for HepG2 treated with SCDs, laser irradiation induced only 25% death, proving that the targeting peptides results in high specificity to cancer cells. PTT on both cell lines with SCDs modified with only RGD (SCDs-RGD) showed no significant difference between cancer and normal cell lines. This finding further proved that RGD only has cancer cell targeting effects, mitochondria-targeting peptide was the major cause for improved PTT efficacy. The mechanism underlying the strengthened photothermal efficiency of SCDs-MT was elucidated by JC-1 probe, and it was observed that the mitochondrial membrane potential of HepG2 cells was lost. This result proved that cancer cell apoptosis was caused by the mitochondria-targeting of SCDs-MT, through disrupting mitochondrial functions. Moreover, real time dynamics of cellular molecules during the process were further evaluated by surface-enhanced Raman spectroscopy and it was revealed that the generated hyperthermia leads to structural changes in lipids, proteins and DNA, and then induces cell death.

**FIGURE 3:**
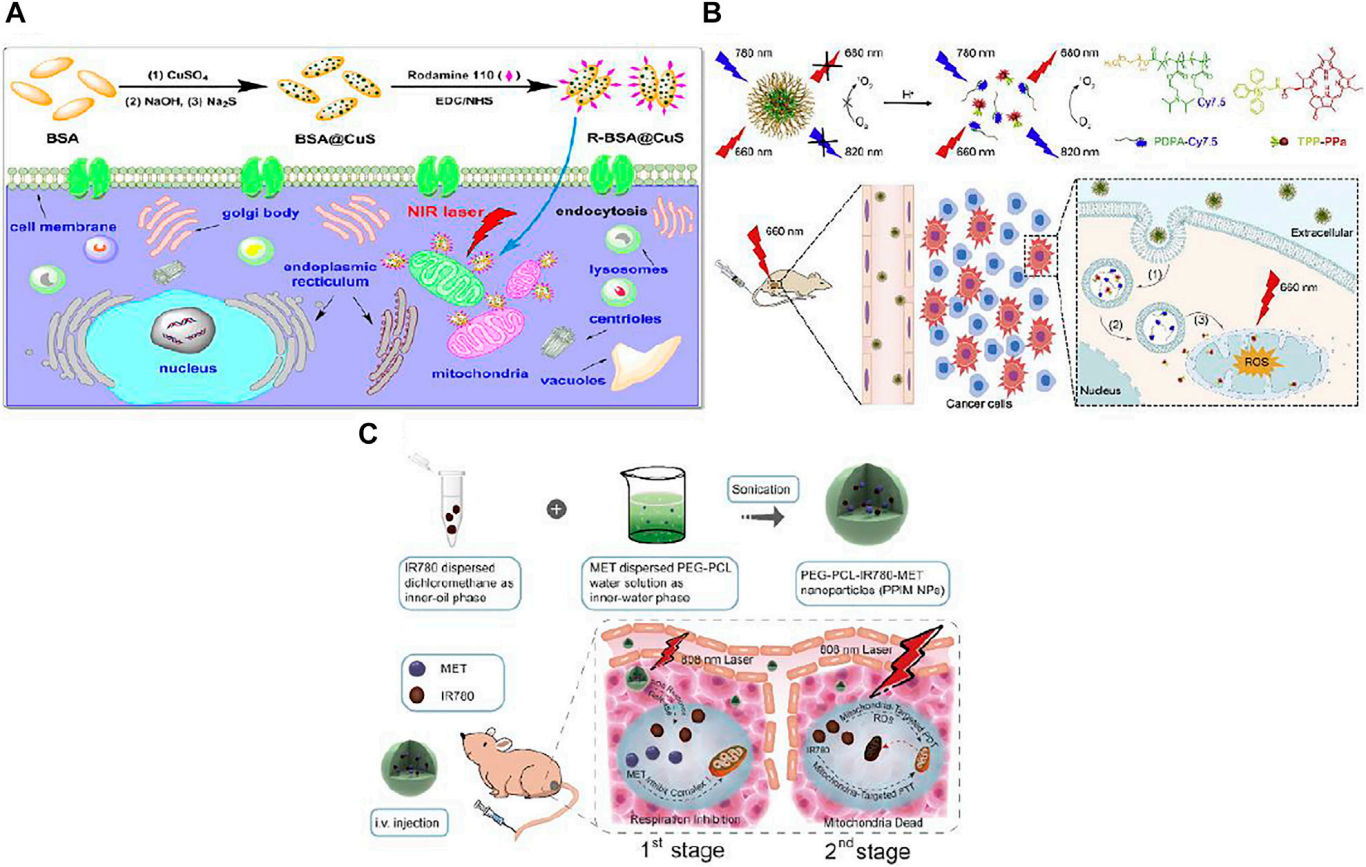
**(A)** CuS nanoparticles were *in situ* synthesized in BSA templates with the conjugation of rhodamine 110 dyes to prepare mitochondria-targeted and photothermal R-BSA@CuS nanocomposites, resulting in an enhanced photothermal therapeutic efficacy of cancer cells *via* destroying their mitochondria and weakening their heat tolerance. Reproduced with permission from Tong et al. (2021). Copyright (2021) Wiley-VCH. **(B)** Schematic illustration of M-TPPa for dual-stage precisely targeting of early endosome and mitochondria to amplify photodynamic therapy, mainly concludes three steps: (1) M-TPPa keepss silent in blood circulation, and can be quickly disassembled in the acidic environment of early endosome (pH 6.0–6.2) to activate the fluorescence signals of dye-conjugated polymer and photoactivity of photosensitizer upon endocytosis; (2) the released cationic TPPa molecules rapidly diffuse into cytoplasm; (3) the cytosolic TPPa targets mitochondria rapidly and efficiently to trigger mitochondria-related cell apoptosis for the enhanced antitumor efficacy under irradiation with 660 nm laser. Reproduced with permission from Qi et al. (2019). Copyright (2019) Elsevier. **(C)** Schematic illustration of the design, synthesis and phototherapeutic functions of PEG-PCL-IR780-MET NPs. Such two-stage phototherapy first inhibited mitochondrial respiration (1st stage for manipulating tumor hypoxia microenvironment) and subsequently attacked the mitochondria of tumor cells (2nd stage for eradicating primary tumor). Reproduced with permission from Yang et al. (2020). Copyright (2020) Elsevier.

PTT efficacy is often hindered by heat resistance of cancer cells resulted from upregulation of heat shock proteins (HSPs) (Li et al., 1995). HSPs are a group of proteins overexpressed when cells encountering heat stress so as to protect cells from hyperthermic damage (Gao et al., 2020). One of the major functions of mitochondria is ATP production to supply cells with energy sustaining life circles, which is also essential for HSPs expression. Besides, because the genes encoded by mitochondria contribute to the evolution of heat tolerance of eukaryotic cells, the functions of mitochondria can be interfered by metabolic and genetic methods to interfere with the heat tolerance of tumor cells. Therefore, disrupting the mitochondria function may down-regulate HSPs, which then further alleviate heat tolerance of tumors resulting in synergistic amplification in PTT efficacy. In this point, a neat construction of copper sulfide (CuS) *in situ* synthesized within bovine serum albumin (BSA) templates then conjugating with rhodamine-110 was obtained for elevated PTT (Tong et al., 2021). The prepared photothermal BSA@CuS nanocomposites possess high efficiency (42.0%) of photothermal conversion while rhodamine-110 as mitochondria directing moiety merit in its self-fluorescence for imaging convenience. The resulting R-BSA@CuS can be easily uptaken by MCF-7 cells and enriched in mitochondria. The viabilities of MCF-7 cells treated with R-BSA@CuS under 2.4, 3.2 W cm^−2^ 808 nm NIR irradiation was significantly lower than that of cells treated with non-targeting BSA@CuS and control, especially under 3.2 W cm^−2^ NIR exposure (with a significant difference of ***p < 0.001). Therefore, mitochondria-targeted R-BSA@CuS exhibited a remarkable enhancement of anticancer efficacy. The mechanism of the proven enhanced PTT of JC-1 assays was mainly ascribed to mitochondria damage with mitochondria membrane depolarization. Besides, HSP90 western blot assays showed that the expression of HSP90 was inhibited after R-BSA@CuS directly destroying mitochondria. Therefore, R-BSA@CuS, when compared to its non-targeting counterpart BSA@CuS, accumulated in mitochondria and generated much higher localized temperature to induce much severer mitochondria damage under the same NIR laser exposure, meanwhile suppressed the heat tolerance of cancer cells, thus more efficiently induced cell death (Figure 3A). The effect was also evaluated in 3D multicellular MCF-7 tumor spheroids that R-BSA@CuS nanocomposites could penetrate tumor spheroids and also showed amplified spheroids growth imbibition when compared to non-targeting BSA@CuS.

### Mitochondria-targeted Nanomedicine for PDT

For PDT, a variety of active photosensitizers can transfer energy to nearby oxygen molecules to generate excess ROS when exposed to photo irradiation. The resulting ROS then oxidizes cellular compositions including lipids, proteins, DNA and ultimately kill cells upon photo excitation. When directing photosensitizer in or around mitochondria area, the over ROS produced beyond the tolerance of mitochondria would oxidize mtDNA, then result in mitochondria membrane rupture and eventually cell apoptosis. Since mitochondria are more susceptible than other intracellular compartments, a given amount of ROS enriched in mitochondria causes much severe cytotoxicity.

Ruthenium (Ru) complexes have drawn great research interest as PDT agents because of their unique optical features. Biocompatible and biodegradable blood plasma protein human serum albumin (HSA) was chosen as backbone with poly(ethylene oxide) (PEO-2000) side chain to be modified with TPP, to improve its aqueous solubility and as mitochondria-targeting group (Chakrabortty et al., 2017). The aniline-modified Ru complexes was finally conjugated to the backbone and to obtain cHSA-PEO-TPP-Ru. cHSA-PEO-TPP-Ru exhibited improved photostability and about 8-fold enhanced singlet oxygen generation when compared to free Ru complex (Ru1) with equivalent concentration of Ru. After incubated with HeLa cells for 6 h and then irradiated with 470 nm LED light source, ∼20 mW cm^−2^ for 5 min, cHSA-PEO-TPP-Ru showed 220-fold increase in cytotoxicity compared to free Ru1, with corresponding IC50 value of 34.9 ± 2 nmol and 7.7 ± 1.3 μmol. Also, the nanomedicine exhibited efficient inhibition of colony growth of acute myeloid leukemia (AML) leukemic cell line, OCI-AML3, compared to normal bone marrow cells. The enhanced phototherapy of the nanomedicine was considered to be attributed to the following aspects: high Ru loading capacity, enhanced cellular uptake efficiency in addition to localization in mitochondria.

The therapeutic efficacy of mitochondria-targeted PDT nanocarrier has been hindered because of their difficulties to escape from endosome and the damage to other normal tissues. Therefore, pH-activatable nanoparticle with dual stage targeting capacity of early endosome and mitochondria was developed in order to tackle this issue (Qi et al., 2019). A pH-responsive fluorescent copolymer, poly(ethylene glycol)-b-poly(2-(isopropylamino) ethyl methacrylate) (mPEG-b-PDPA) was conjugated with Cy7.5 and mitochondria-targeted photosensitizer, (triphenyl) phosphonium conjugated pyropheophorbide-a (TPPa) to finally form M-TPPa. Because of hetero FRET effect from TPPa to Cy7.5, the resulting M-TPPa was off for the fluorescent signal and photoactivity in neutral pH but obtained drastic fluorescence signal augment and photoactivity recovery in an acidic environment. As a result, M-TPPa does not show fluorescence or photoactivity in blood circulation system via intravenous injection, which is favorable for the alleviation of adverse effects on normal tissues. Once M-TPPa was firstly localized in endosome after endocytosis, the acidic environment in endosome made them rapidly disassembled to trigger the activation of the fluorescence signals. The released photosensitizer, TPPa then escaped from endosome to accumulate in mitochondria due to directing TPP group (Figure 3B). Finally, TPPa inhibits cancer cell by generating ROS near mitochondria upon laser exposure. After exposure to 660 nm laser the M-TPPa treated cells showed significantly higher ROS generation compared to control, laser only, or M-TPPa without laser exposure groups. And the ROS signal is superimposable with PPa, suggesting the specific mitochondria-targeted ROS generation. The *in vitro* cytotoxicity was evaluated after HO8910 ovarian cancer cells were co-incubated with M-TPPa, TPPa, PPa, or PDPA. PDPA+Laser or TPP+L showed no obvious cytotoxicity, both functional polymer and mitochondria-targeted ligand had no phototoxicity. Compared to PPa group, M-TPPa under 660 nm laser irradiation (M-TPPa + L) or TPPa under NIR light irradiation (TPPa + L) demonstrated amplified cancer cell cytotoxicity, which was attributed to highly efficient mitochondria-targeted photodynamic effects. The PDT mechanism was also elaborated that M-TPPa phototherapy induces efficient MMP loss, and further disrupts mitochondria function and initiates intrinsic cancer cell apoptosis. This dual-stage precisely mitochondria-targeted M-TPPa provides augmented photodynamic anticancer efficacy.

In addition to ruthenium and pyropheophorbide-a, some nanoscale metal-organic layer (nMOL) complexes are also employed as photosensitizers. Zinc(II)-2,3,9,10,16,17,23,24-octa(4-carboxyphenyl)phthalocyanine (ZnOPPc) photosensitizer was conjugated on the secondary building units (SBUs) of a Hf_12_ nMOL to obtain ZnOPPc@nMOL (Nash et al., 2021). This ZnOPPc@nMOL has innate mitochondria-targeting capability and exhibited high PDT efficiency with greater than 99% tumor growth inhibition on mouse models of colon cancer.

### PDT Combined with PTT for Synergistic Effects

In more cases, mitochondria-targeted PDT and PTT are combined for synergistic effects, producing oxidizing stress and hyperthermia in or around mitochondria concurrently, herein opening up possibilities to maximize the phototherapeutic effects. Photothermal and photodynamic properties can be combined on one material. Organic nanomaterials are one of candidates to consider because of their diverse structures for realizing versatile functions. Diketopyrrolopyrrole-based molecule, 2,5-bis(6-bromohexyl)-3,6-bis(5-(4-(diphenylamino)phenyl)thiophen-2-yl)-2,5-dihydropyrrolo[3,4-c]pyrrole-1,4-dione (DPP) was firstly designed and synthesized by Li and colleagues (2020). One or two imidazole groups were introduced to the DPP molecule to render the mitochondria-targeting ability, named as DPP+ and DPP2+ respectively. All the three derivatives can self-assemble to form nanoparticles (DPP NPs, DPP+ NPs and DPP2+ NPs). The photothermal conversion efficiency of DPP NPs, DPP+ NPs and DPP2+ NPs was 32%, 34.5%, and 35%, under 0.3 W cm^−2^ 635 nm laser irradiation. All the three compounds can produce singlet oxygen under 635 nm laser irradiation, as characterized by 1,3-diphenylisobenzofuran (DPBF) tests. Among these three compounds, DPP2+ NPs with two imidazole groups showed enhanced cellular uptake and the most accumulation in mitochondria, when comparing with other two NPs. *In vitro* viability tests showed that in the dark, cell viabilities were maintained more than 90% after cells are co-incubated with all three NPs, suggesting good biocompatibility of the three NPs. However, after cells were exposed to 0.3 W cm^−1^ 635 nm laser for 5 min, DPP+ and DPP2+ showed cytotoxicity, with DPP2+ exhibiting greater cytotoxicity than the DPP+, while DPP showed negligible cytotoxicity. In order to investigate the major factors for cell inhibition, they ruled out the photothermal effect by cooling down the experiment environment temperature to 4°C and photodynamic effect by introducing NaN_3_ to release nitrogen for ROS scavenging. It was found that it was the synergistic effect of PTT and PDT that caused cell inhibition, while PDT played the major role in the treatment. DPP2+ exerted cytotoxicity at a concentration as low as 5 μmol under a low irradiation intensity (0.3 W cm^−1^) for only 5 min. In addition, HeLa cells treated with DPP2+ NPs produced more cellular singlet oxygen than DPP+ and DPP NPs, demonstrating better photodynamic effects of DPP2+. The therapeutic mechanism was attributed to MMP loss as proved by JC-1 probe tests, suggesting that directing PDT and PTT to mitochondria leads to enhanced therapeutic efficacy. For *In vivo* test, DPP2+ NPs have been demonstrated significant inhibition on subcutaneous mouse cervical U14 tumors, coated by F127 to improve their solubility and stability, for both intravenous and intratumoral injections. This specially designed compound works effectively and efficiently as it has PDT, PTT and mitochondria-targeting capability all together in one molecule.

IR780 is also the type of compound that integrates PDT and PTT attributes in one molecule. In order to combat relapsed and refractory tumor, a strategy of inhibiting mitochondria respiratory and damaging mitochondria was developed by Yang et al. (2020). Tumor hypoxia microenvironment can reduce the therapeutic effect on tumors. In order to reduce oxygen consumption, IR780 and metformin were encapsulated in amphipathic poly(ε-caprolactone)-poly(ethylene glycol) (PEG-PCL) liposomes to form PEG-PCL-IR780-MET (P-P-I-M) nanoparticles. Metformin can inhibit the activity of complex I in the mitochondrial electron transport chain, and P-P-I-M exhibited similar inhibition to mitochondrial complex I and depolarized mitochondria, proved by MMP assay using JC-1 kits. Thus P-P-I-M depressed the cell respiration and reduced the cancer cell oxygen consumption, while showed almost no cytotoxicity to human gastric cancer cell line MKN-45P cells. After irradiated with NIR and released from P-P-I-M NPs, IR780 were localized in mitochondria due to its lipophilic cationic feature (Figure 3C). After exposed to 808 nm laser, cancer cells treated with P-P-I-M showed greatest ROS production and least viability when compared to that treated with PEG-PCL-IR780 (P-P-I) and PEG-PCL-MET (P-P-M) (16.7, 45.3, 69.6%, respectively). This result proved that by combining the hypoxia alleviating effect by MET and mitochondria-targeted capability of IR780, PDT efficacy of P-P-I-M nanoparticles against MKN-45P cells was magnified. PDT or PTT might be attenuated by hypoxia in tumor microenvironment (TME), while MET could weaken the level of hypoxia. Besides, the IR780 concurrently endowed with NIR and photoacoustic (PA) imaging capability which is favorable for NIR/PA bimodal imaging, and could further guide the therapy. *In vivo* experiment also exhibited similar results that P-P-I-M under the exposure of 808 nm laser inhibit tumor growth and recurrence by overcoming tumor hypoxia and mitochondria-targeted synergistic PDT and PTT therapy.

Another strategy in this criterion is by employing PDT to destroy mitochondria and inhibit the ATP generation and overexpression of HSPs, thus achieving amplified PTT. A self-mineralized photothermal bacterium (PTB) hybridizing with mitochondria-targeted metal-organic framework was designed and cause mitotoxic effects by PDT to enhance PTT, by down regulating HSPs (Chen et al., 2020). Electrochemically active and facultative anaerobic bacterium *Shewanella oneidensis* MR-1 (*S. oneidensis* MR-1) reduces sodium tetra-chloropalladate (Na_2_PdCl_4_) to palladium nanoparticles (Pd NPs) on its surface to form photothermal bacterium (PTB), with their bioactivities maintained. This PTB renders good tumors-targeting ability because of the hypoxia chemotaxis and together with excellent photothermal properties in the NIR region resulted from the addition of Pd NPs as a photothermal agent. In order to improve the PTT efficiency of PTB, Zeolitic imidazole framework-90 (ZIF-90) loaded with photosensitizer methylene blue (MB) was further conjugated on the surface of living PTB. The PTB@ZIF-90/MB was prepared via acid-sensitive imine bond. Therefore, when PTB@ZIF-90/MB reached the acidic tumor site, ZIF-90/MB was shed from the PTB surface due to acid-sensitive imine bond breakage, and endocytosis by cancer cells. The ZIF-90 was formed by self-assembling of Zn^2+^ and imidazolate-2-carboxaldehyde (2-ICA), and since ATP has a much stronger coordination between and Zn^2+^ than 2-ICA, ZIF-90/MB can be degraded by ATP through competing Zn^2+^ binding way. Thus, upon being internalized by cells, ZIF-90/MB can selectively release MB in the mitochondria, where ATP are enriched. The released MB produced large amount of singlet oxygen under 660 nm light exposure for 5 min. Therefore, ZIF-90/MB augmented the mitochondria oxidative stress by specifically targeting mitochondria and generating oxidative singlet oxygen under laser irradiation, which leads to mitochondrial dysfunction by disrupting mitochondrial redox homeostasis. This mitochondria injury in the end resulted in down-regulating HSPs expression, as proved by western blotting. Furthermore, PTB was hybridized with ZIF-90/MB to obtain PTB@ZIF-90/MB for extracellular PTT. The amplified photothermal efficacy of PTB@ZIF-90/MB was demonstrated by 3-(4,5)-dimethylthiazol-2-yl)-2,5-diphenyl-tetrazolium bromide (MTT) assays to show that low concentration (OD_600_ = 0.05) of PTB@ZIF-90/MB resulted in amplified cancer cell inhibition (approximate 70%) after combining 660 nm light and 808 nm laser exposure. *In vivo* experiment also demonstrated augmented PTT efficacy of PTB@ZIF-90/MB. And the mechanism of the enhanced PTT efficacy was elucidated down regulation of two typical HSPs of HSP70 and HSP90 by immune fluorescence staining. As a whole, the PTT platform based on PTB@ZIF-90/MB with the advantages of tumor selective targeting capacity and inhibiting tumor heat resistance, has achieved significant therapeutic effects on tumor.

In summary, in contrast to a certain amount of heat produced by nanoagents acting on the whole cell on average, PTT focused in mitochondria leads to more dramatic temperature elevation, resulting in death of cancer cells with higher photothermal efficacy. Similarly a given amount of ROS generated by nanoplatform photosensitizer is now localized in mitochondria for a remarkable PDT efficacy. In addition, PDT and PTT are also combined for a synergistic effect, enabled by photosensitive and photothermal attributes integrated on one molecule.

## Mitochondria-targeted Nanomedicine for Enhanced CDT

CDT was first proposed by Bu’s group (Zhang et al., 2016; Tang et al., 2019) in 2016, inspired by Fenton reaction originally in environmental science, based on amorphous iron. CDT falls into the criteria of nanocatalytic medicines. In CDT, endogenous chemical energy was employed in the ROS production. CDT takes advantage of the weak acidity of TME as the reaction condition, over expressed H_2_O_2_ as the reaction material, and transitional metal nanomaterials as catalysts, to trigger Fenton or Fenton-like reactions in cancer cells. H_2_O_2_ is catalyzed to produce toxic hydroxyl radical (·OH) and other strong oxidizing active substances, thus tumor cell apoptosis is induced. The ideal environment for Fenton reaction is acidic. However, the pH in mitochondria is 8.0, which is slightly alkaline and not suitable for Fenton reaction. Therefore, the work of mitochondria-targeted nanomedicine for enhanced CDT that was already reported up to now does not move Fenton reaction to mitochondria area, it involves depleting glutathione (GSH) which is overexpressed in mitochondria and as an intracellular antioxidant that protect cells from oxidization stress, attenuating the effects of CDT (Shen Y. et al., 2020). Thus, depletion of GSH causes decrease of the free radical scavenging, and increases the therapeutic effects of CDT.

**FIGURE 4:**
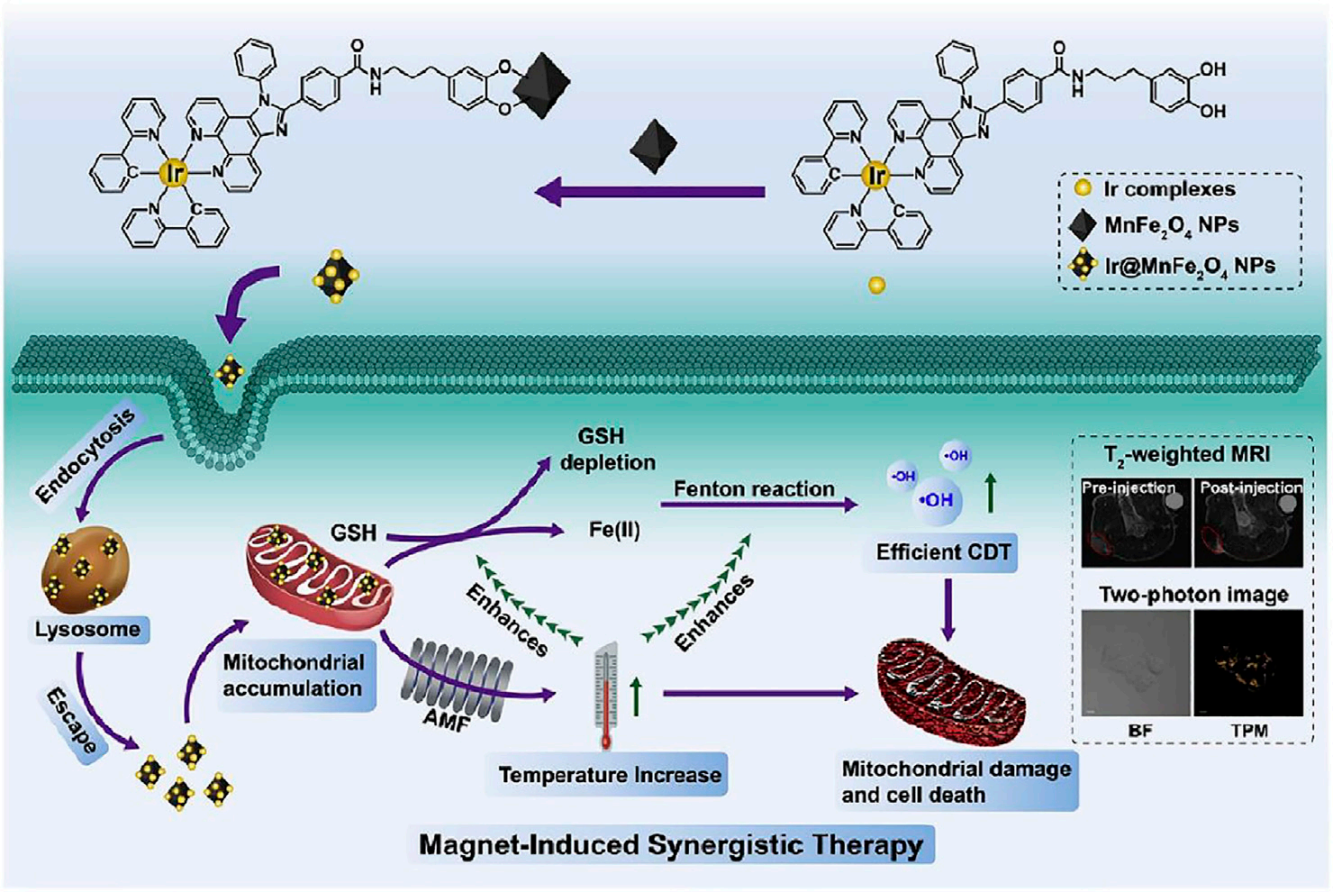
The synthesis of Ir@MnFe2O4 NPs, and a schematic representation of Ir@MnFe2O4 NPs as mitochondria-targeting magnetothermogenic nanozymes for magnet-induced synergistic therapy. Reproduced with permission from Shen J. et al. (2020). Copyright (2020) Elsevier.

In this work, a magnetothermogenic nanozyme, Ir@MnFe_2_O_4_ NPs, was designed with a lipophilic cationic cyclometalated iridium(Ⅲ) complex (Ir) for mitochondria-targeting and two-photon fluorescent imaging, together with MnFe_2_O_4_ causing local temperature rise upon the exposure of an alternating magnetic field (AMF). In addition, MnFe_2_O_4_ plays a role in catalyzing nanozyme. After endocytosed by tumor cells, Ir@MnFe_2_O_4_ NPs accumulate in mitochondria with the help of cyclometalated iridium (Ⅲ) complex on their surface. Fe(Ⅲ) was reduced to Fe(Ⅱ) by overexpressed GSH in mitochondria area and the resulting Fe(Ⅱ) catalyzes H_2_O_2_ to hydroxyl radicals *via* Fenton reaction. Meanwhile, the reduction of Fe(Ⅲ) results in the depletion of GSH, which further a decrease in the radical scavenging. At the same time, the magnetothermo Ir@MnFe_2_O_4_ NPs caused a localized hyperthermia in mitochondria when exposed AMF. Mitochondria are highly susceptible to heat, therefore, this generated heat focused in mitochondria destroys mitochondria, further destruct cancer cells. In addition, the intracellular temperature rise accelerates the conversion of Fe(Ⅲ) to Fe(Ⅱ) by GSH. As a result, the rate of cytotoxic ·OH generation is elevated as well as GSH consumption, leading to disruption of cellular redox homeostasis and greater susceptibilities of cells to hyperthermia. Thus, the enhancement of therapeutic efficacy was a synergistic effect of targeting magnetic hyperthermia therapy in mitochondria and accelerating CDT and consumption of GSH by generated heat (Figure 4). The *in vitro* cell inhibition activity was determined by Calcein-AM/PI test. HeLa cells treated with 0.4 mg ml^−1^ Ir@MnFe_2_O_4_ while exposing to AMF showed PI positive while the cell viabilities maintained in control, control + AMF, or NP only groups. The inhibition effect can be attributed to MMP loss proved by JC-1 probe. Moreover, in MTT assays, the viability of cells treated with Ir@MnFe_2_O_4_ NPs (0.4 mg ml^−1^) with the application of an AMF for 40 min was 14%, while the viability of cells treated with γ-Mn_0.2_Fe_1.8_O_3_ NPs without Ir complexes (2.25 mg ml^−1^) in AMF for 45 min was about 62%. These results together suggest that both AMF exposure and mitochondria-targeting are essential for amplified cell cytotoxicity induced by the Ir@MnFe_2_O_4_ NPs. The synergistic therapy effect was also evaluated *in vivo*.

## Mitochondria-targeted Nanomedicine for Enhanced SDT

Besides chemical and optical means, acoustic wave also enables ROS generation. Sonosensitizers are activated to produce ROS by ultrasound (US) irradiation and provide underlying mechanism for SDT. In contrast to other ROS-mediated cancer therapies like PDT, SDT stands out in deep tissue penetration. US is a mechanical wave, thus SDT has reduced invasiveness and is nonradioactive. Although the exact mechanism of SDT is still under investigation, it is generally believed that sonosensitizers can transfer energy to nearby oxygen molecules upon US introduction, which then leads to ROS generation (Zhang et al., 2019). The ultrasonic cavitation effect of US is considered to be the major cause for sonoluminescence and pyrolysis process, which then caused sonosensitizers activation. When sonosensitizer is directed to mitochondria, the produced ROS upon US irradiation are focused in mitochondria which are sensitive to ROS. Thus, resulting augmented therapeutic efficacy combating tumor tissues.

In a mitochondria-targeted SDT, Au nanocrystals were decorated on the active (001) facet edge of TiO_2_ nanosheets (NSs) to prepare Au-TiO_2_ NSs (Cao et al., 2019). The narrowed band gap of Au-TiO_2_ NSs together with better transferring of interfacial electron in the composites by Au, resulted in higher ROS quantum yield than pure TiO_2_. TPP and AS1411 aptamer (A) were further conjugated to the sonosensitizer Au-TiO_2_ for mitochondria- and cancer-cell-targeting purposes respectively. As-prepared Au-TiO_2_-A-TPP sonosensitizer could easily be uptaken by cancer cells and enriched in the mitochondria with good biocompatibility and long circulation time. The viability of MCF-7 cells treated with both cancer-cell- and mitochondria-targeting Au-TiO_2_-A-TPP decreased to 9.8% which was much lower than those of cells treated with non-targeting Au-TiO_2_ (37.5%) and non-mitochondria-targeting (only cancer-cell-targeting) Au-TiO_2_-A (57.6%) in response to the same intensity of US introduction. This result proved that AS1411 aptamer facilitating sonosensitizer to targeting cancer cells contributed to elevated therapeutic efficacy compared to non-tumor-targeting sonosensitizer, however the therapeutic efficacy was less effective than sonosensitizer concurrently modified by TPP and aptamer with cancer cell and mitochondria dual-targeted capability. Accordingly, cells treated with Au-TiO_2_-A-TPP produced higher ROS amount under US irradiation than cells treated with Au-TiO_2,_ Au-TiO_2_-A as measured by DCFH-DA probe. Similarly, Au-TiO_2_-A-TPP treated group exhibited better *in vivo* tumor suppression effects than Au-TiO_2_ and Au-TiO_2_ -A group.

**FIGURE 5:**
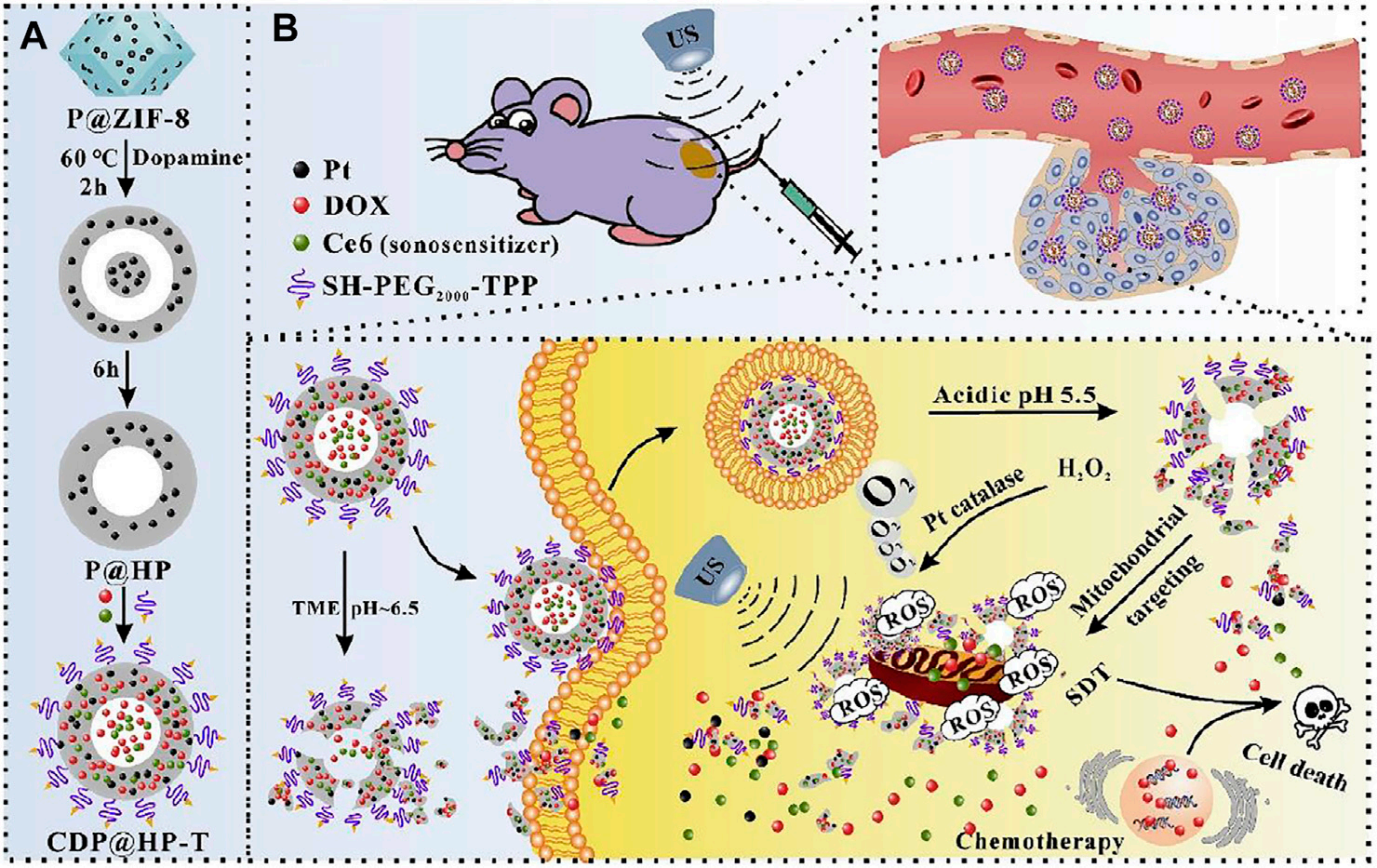
Schematic illustration of the synthesis route of CDP@HP-T **(A)** and chemo-sonodynamic combined therapy. **(B)** Reproduced with permission from An et al. (2020). Copyright (2020) Elsevier.

Mitochondria-targeted SDT like other therapies was also coupled with other different types of therapies to achieve augmented efficacy. A nanocarrier with catalytic function to increase ROS and pH-responsive drug release was designed combining SDT with chemotherapy for enhanced mitochondria-targeted antitumor effects (An et al., 2020). Hollow polydopamine nanoparticles was embedded with platinum nanoparticles, co-loaded with DOX and Ce6, and finally modified with TPP. The prepared CDP@HP-T could easily accumulate in the tumor with excellent biocompatibility. The CDP@HP-T nanoparticle could release DOX and Ce6 in slightly acidic TME and/or subcellular lysosome. After cellular uptake, the released DOX kill tumor cells by destroying the nucleus. As TPP directs the nanocarrier to mitochondria, Pt nanoparticles on CDP@HP-T catalyzed the decomposition of endogenous H_2_O_2_ to *in situ* produce O_2_ near mitochondria, supplying the source of ROS and alleviating hypoxia of tumor (Figure 5). While Ce6 is a sonosensitizer that produce ROS upon US treatment. All these conditions contributed to amplified ROS production in mitochondria. Combining chemotherapy and SDT using CDP@HP-T could lead to amplified effects than a single therapy. Therapeutic efficacy of mitochondria-targeted CDP@HP-T nanoparticle was better than that of non-targeted CDP@HP group under same US irradiation, while their difference was more significant at higher concentration. Demonstrating targeting mitochondria conferred by the targeting capability of TPP that enhanced the cancer cell cytotoxicity. *In vivo* also showed mitochondria-targeting CDP@HP-T exhibited better tumors suppression than non-targeting CDP@HP by combined SDT and chemotherapy.

## Mitochondria-targeted Nanomedicine for Enhanced RDT

**FIGURE 6:**
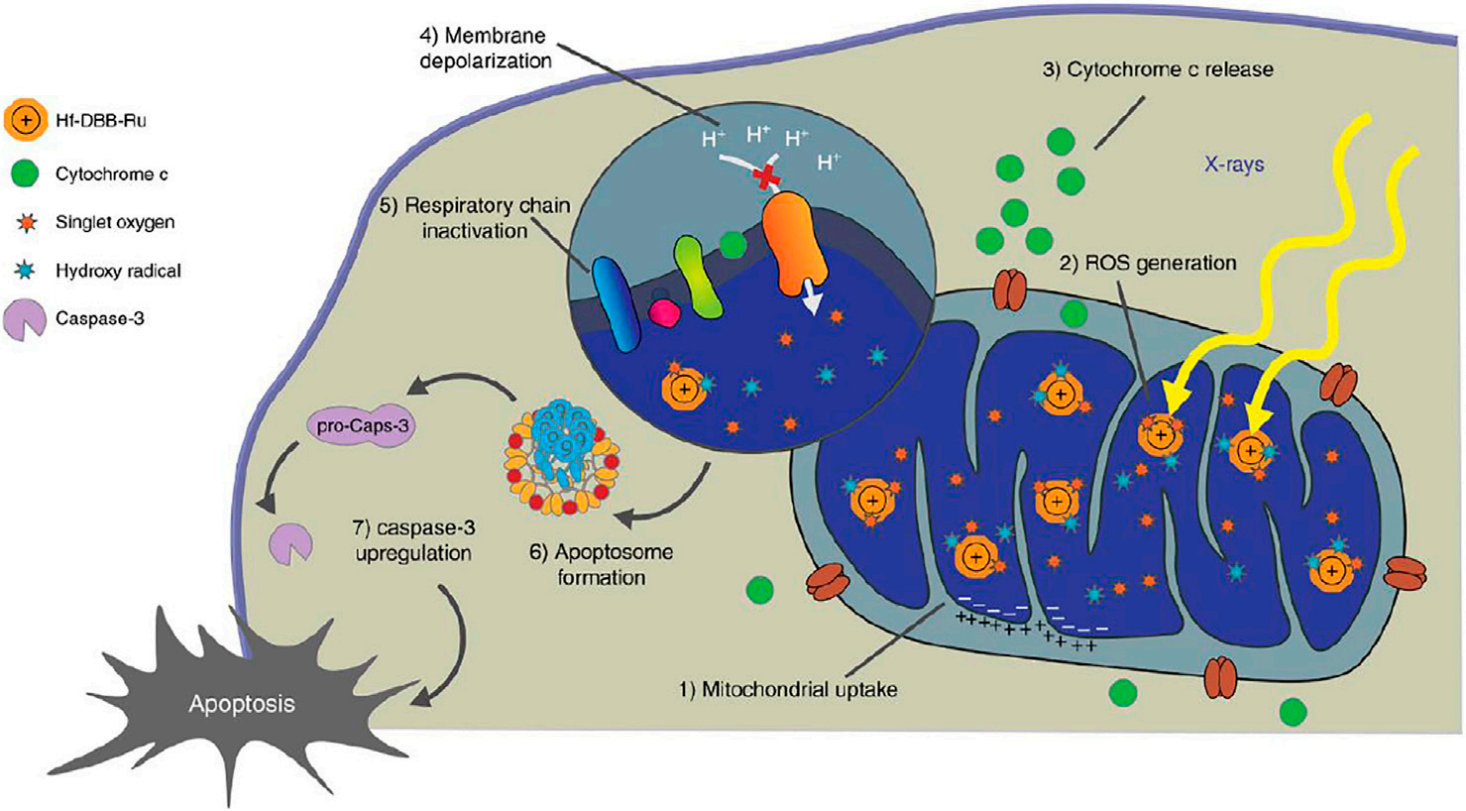
Mitochondria-targeted RT-RDT mediated by Hf-DBB-Ru. Hf-DBB-Ru was internalized by tumor cells efficiently and enriched in mitochondria due to dispersed cationic charges in the nMOF framework. Hf_6_ SBUs preferentially absorb X-rays over tissues to enhance RT by sensitizing hydroxyl radical generation and enable RDT by transferring energy to Ru(bpy)_3_^2+^-based bridging ligands to generate singlet oxygen. The RT-RDT process trigger mitochondrial membrane potential depolarization, membrane integrity loss, respiratory chain inactivation, and cytochrome c release to initiate apoptosis of cancer cells. Reproduced with permission from Ni et al. (2018). Copyright (2018) Nature.

Another means of ROS generation is by X-ray irradiation, through specific nanostructures, like Hf-porphyrin nanoscale metal-organic frameworks (nMOFs) (Ni et al., 2018). Hf-porphyrin nMOFs produced hydroxyl radicals from the Hf SBUs and singlet oxygen from the porphyrin photosensitizers, when exposed to X-ray. These class of nMOFs have shown their advantages in nanomedicine applications as the ordered crystalline structure and innate porosity of nMOFs prevent them from self-quenching, and facilitate the diffusion of ROS. RDT is superimposed with RT, achieving double effects concurrently when exposed to a given amount of X-ray. A mitochondria-targeted Ru-based nMOFs, Hf-DBB-Ru, for enhanced RT-RDT was reported by Ni et al., while DBB-Ru represents Bis (2,2’-bipyridine) (5,5’-di(4-benzoato)-2,2’-bipyridine) ruthenium (II) chloride. By incorporating Ru(bpy)_3_^2+^ photosensitizer into the framework, Hf-DBB-Ru presents a cationic UiO topology and high potential for mitochondria-targeting. Upon low dose of high penetrating X-ray irradiation, Hf-DBB-Ru achieved RT-RDT, by generating hydroxyl radicals from the Hf_6_ SBUs and singlet oxygen from the DBB-Ru photosensitizers (Figure 6). Similar with PDT, CDT and SDT, mitochondria-targeted RDT focuses the ROS produced by X-ray photosensitizer in the mitochondrial region to cause greater damage to cancer cells.

Similar nMOF was also incorporated with an inhibitor of the immune checkpoint indoleamine 2,3-dioxygenase (IDOi) to achieve RT-RDT combined with immunotherapy for both local and distant tumor inhibition (Lu et al., 2018).

## Mitochondria-targeted Nanomedicine for Combined Immunotherapy

In novel cancer therapy modalities, cancer immunotherapy represents the most innovative approach (Couzin-Frankel, 2013). Immunotherapies for cancer are a range of treatments that activate the immune system to fight cancer. Normally, the immune system can recognize and eliminate tumor cells in the TME. But in order to survive and grow, Tumor cells develop different strategies so that the immune system is inhibited and cannot kill tumor cells. As a result, cancer cells can survive in various stages of antitumor immune responses. The process is the immune escape of tumor cells. Tumor immune cycles include the following steps after tumor cells are killed by other therapies like chemotherapy, etc.: *1*) tumor associated antigens (TAAs) release, *2*) tumor antigen presentation by dendritic cells, *3*) activation of effector T cells, *4*) T cells searching, tracking and migrating to tumor tissues, *5*) T cells infiltration in tumor tissue, *6*) specific recognition of tumor cells by T cells, *7*) tumor cells killing (Shao et al., 2015). Any malfunctions in these steps can lead to the failure of tumor immune cycle and immune escape. Different tumors can avoid being recognized or attacked by the immune system through different mechanisms, for example, by expressing a specific immunosuppressive protein on their surface. This inhibition of immune system results in immune tolerance, and even promote the occurrence and development of tumors. So, cancer immunotherapy is a treatment to control and eliminate tumor by restarting and maintaining the tumor immune cycle and restoring normal antitumor immune responses. Cancer immunotherapy takes the advantages of monoclonal immune checkpoint inhibitors, therapeutic cancer vaccines, adoptive cell therapy (Cheng et al., 2009). A number of tumor immunotherapy medicine has been approved by US Food and Drug Administration (FDA) for clinical use or under clinical trials. Immunotherapy is often combined with other treatment modalities directing to mitochondria to facilitate improved effects. The efficacy enhancement of mitochondria-targeted combined immunotherapy by nanomedicine is often attributed to elevated treatment effects of other therapies when targeting to mitochondria. Their improved efficacy of killing tumor cells leads to more tumor antigen released, which is the initial and essential step in the tumor immune cycle, resulting in a cascade amplification domino-like effect.

A multistage nanomedicine with tumor and mitochondria-targeting capability was designed for enhanced PDT and combined immunotherapy (Yang et al., 2018). The nanoplatform was constructed on hollow silica nanoparticles loaded with H_2_O_2_ enzyme, catalase (CAT) in cavities, and photosensitizer Ce6, modified with mitochondria-targeting (3-carboxypropyl) triphenylphosphonium bromide (CTPP). The resultant nanoparticles were coated with charge convertible polymer either dimethylmaleic anhydride (DMMA)-PEG (DPEG) to form pH-responsive nanocarrier or succinic anhydride (SA-PEG) (SPEG) to form pH-inert nanoparticles as a control. For DPEG coated nanoparticle (CAT@S/Ce6-CTPP/DPEG), when pH changes from 7.4 (corresponds to normal tissue pH) to slight acidic environment 6.8 (corresponds to TME), the charge was rapidly converted from negative to positive, while for SPEG coated nanoparticle (CAT@S/Ce6-CTPP/SPEG), the charge remained negative during this pH change. This conversion from negative to positive charge would allow endosome escape of the nanoparticles and enhance the cellular endocytosis in tumor environment. After cellular uptake by cells, TPP ligand directed the nanoplatform to mitochondria, and CAT decomposed endogenous H_2_O_2_ to oxygen within tumor cells, alleviating tumor hypoxia. The produced oxygen then generated excess singlet oxygen by photosensitizer Ce6 when exposed to photo irradiation. Cellular cytotoxicity of mitochondria-targeted CAT@S/Ce6-CTPP/DPEG was significantly augmented compared to non-targeted CAT@S/Ce6/DPEG upon PDT due to the mitochondria-targeting capability of CTPP. *In vivo* animal tests also demonstrated similar results that best antitumor efficacy of PDT was achieved by CAT@S/Ce6-CTPP/DPEG. And the local PDT treatment with the nanoplatform could not only kill cancer cells but also initiate antitumor immune responses, releasing TAAs from damaged tumor cells afterwards. The released TAAs were processed and presented by professional antigen presenting cells like dendritic cells to activated cytotoxic T lymphocytes (CTLs). The antitumor activity of CTLs were enhanced by anti-PD-L1 checkpoint blockade. CTLs migrated into non-irradiated distant tumors and induce cellular immunity to metastasis tumor. As a result, the nanoplatform based PDT inhibited local tumor growth as well as distant tumor metastases. The amplified efficacy of PDT combined with immunotherapy was ascribed to targeting PDT to mitochondria resulting in more severe killing effects on tumor cells, leading to more TAAs release.

**FIGURE 7:**
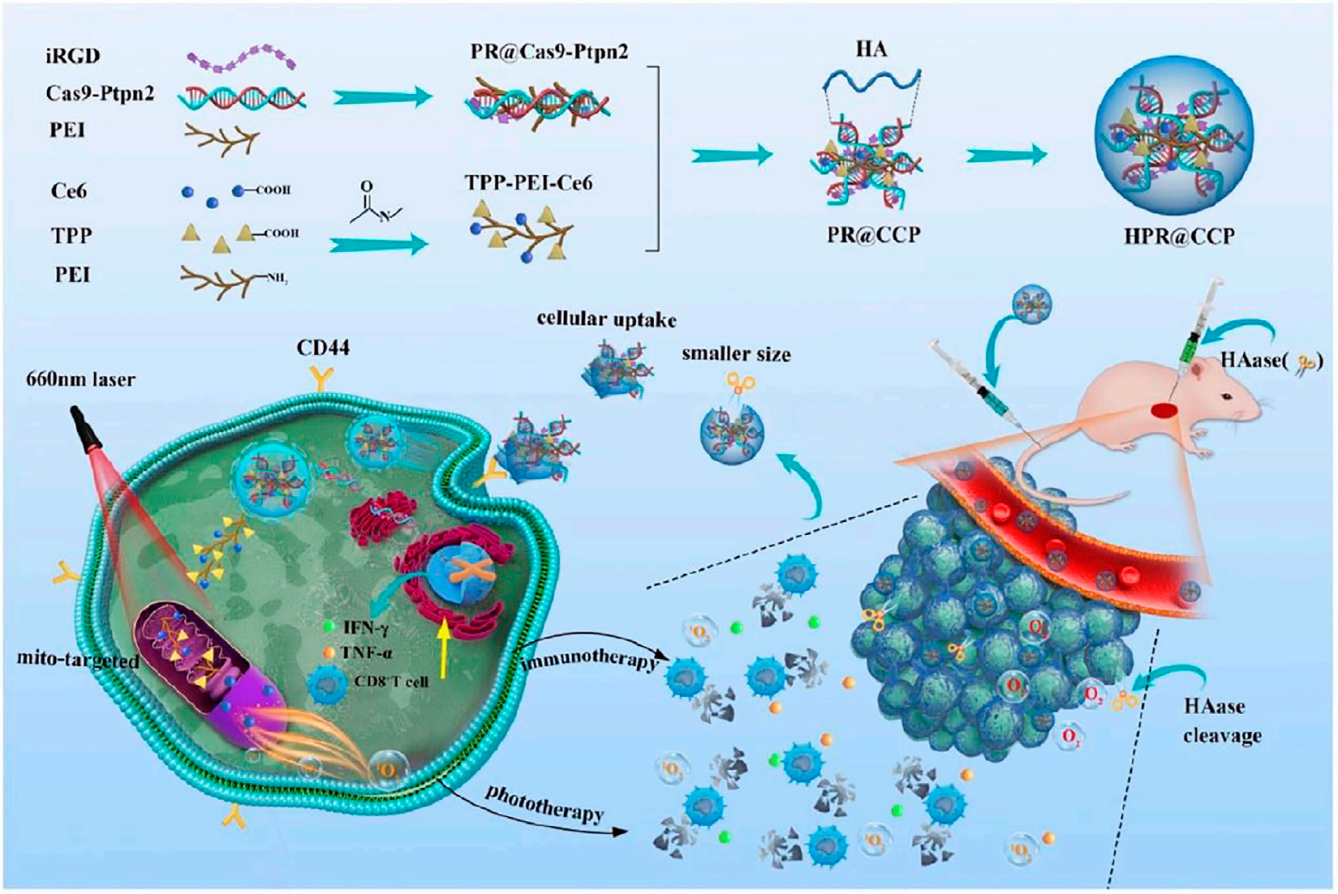
The construction of HPR@CCP nanoparticles and their versatile application in cancer combined therapy. Reproduced with permission from Yang et al. (2020). Copyright (2020) Elsevier.

Yang et al. (2020) reported a Ce6 and CRISPR-Cas9 dual loaded system for a synergistic photodynamic-immunotherapy. The nanosystem was composed of Cas9-Ptpn2 PEI iRGD obtained by electrostatic adsorption of negatively charged hyaluronic acid “shell” and positively charged “core,” which is composed of CRISPR-cas9 system (Cas9-Ptpn2) targeting Ptpn2 gene and modified Ce6 (TPP-PEI-Ce6) targeting mitochondria. CRISPR/Cas9 (clustered regularly interspaced short palindromic repeats associated protein 9) is gene editing technique. Protein tyrosine phosphatase non-receptor type2 (Ptpn2) is a phosphatase (Figure 7). First, Cas9-Ptpn2 and tumor targeting peptide (iRGD) were bound to PEI *via* electrostatic interaction to obtain PR@Cas9-Ptpn2. Then, the PR@Cas9-Ptpn2 were linked with photosensitizer Ce6 through electrostatic attraction, and Ce6 was conjugated to TPP modified PEI via amide bond to obtain PEI-iRGD@Ce6+Cas9-Ptpn2 (PR@CCP). PR@CCP was coated with hyaluronic acid (HA) to form HA-PEI-iRGD@Ce6+Cas9-Ptpn2 (HPR@CCP) nanoparticles. *In vitro* intracellular ROS generation from PDT was tested by DCFH-DA. Cells treated with HPR@CCP showed strongest fluorescence when compared to control, free Ce6 and TPP-PEI-Ce6 groups, proving the targeting ability of HA which delivered more Ce6 moiety. Mito-SOX, a mitochondrial probe sensitive to mitochondrial superoxide production was employed to explore whether Ce6 targeting the mitochondria was the reason for intracellular ROS increase. Again, compared to other three groups, cells treated with HPR@CCP exhibited strongest fluorescence which is significantly stronger than that in the free Ce6 group, indicating that the intracellular ROS were majorly produced by mitochondria. Thereafter, photo-cytotoxicity of HPR@Ce6 was evaluated higher than that attributed to Ce6 upon laser irradiation, which was consistent with previous ROS generation results. HPR@CCP Cell experiments showed that, HPR@CCP nanoparticles have high transfection efficiency on mouse skin melanoma cells (B16F10). Through active and passive tumor targeting *in vivo*, the accumulation of nanoparticles in tumor led to the decrease of Ptpn2 protein by Cas9-Ptpn2 plasmid in the nanocarrier. As a result, inflammatory cytokines of IFN-γ and TNF-α were upregulated, which promoted the proliferation of CD8^+^ T cells, so as to make tumor more sensitive to immunotherapy. Moreover, by pre-administration of hyaluronidase, hyaluronic acid in the agglomerated extracellular matrix were resolved and the hyaluronic acid “shell” of the nanosystem was shed, which subsequently leads to normalized tumor vasculatures, alleviated hypoxia in the tumor and enhanced therapeutic agents. After PDT immunotherapy, the HPR@CCP resulted in remarkable inhibition of the primary melanoma and metastatic tumor of experimental animals. Mitochondria-targeted modification of Ce6, together with CRISPR/Cas9-Ptpn2 system enabled amplified PDT-immunotherapy.

## Conclusion and Future Prospect

Mitochondria are subcellular organelles of vital importance since they provide energy for eukaryotic cells, control many cellular signaling pathways and regulate a variety of cellular events like differentiation, proliferation, apoptosis and necrosis. Their pivotal functions make them closely related to cancer progress. Thus, mitochondria present an attractive subcellular target in cancer therapy. Various mitochondria-targeted nanomedicines have been developed for chemotherapy, PTT, PDT, CDT, SDT, RDT and combined immunotherapy to improve anticancer therapy efficacy. The mechanism of mitochondria-targeted nanomedicine is to trigger intrinsic cell apoptosis and/or cell necrosis mediated by mitochondria, either by damaging mtDNA, disturbing redox homeostasis, increasing endogenous or exogenous ROS. Mitochondria-targeted chemotherapy works through triggering mitochondria mediated apoptosis and overcoming drug resistance. The underlying mechanism for enhanced mitochondria-targeted PTT is to cause a sharp rise in temperature in mitochondria area, and bring down the excretion of HSPs that protect cells from hyperthermia. Principle of augmented mitochondria-targeted PDT, SDT or RDT lies in increasing the amount of ROS, especially exogenous ROS, in mitochondria *via* different characteristic agents including photosensitizers, sonosensitizers and X-ray photosensitizer. While the reports of improved mitochondria-targeted CDT are rare, since the mitochondrial pH environment is not ideal for Fenton reaction. Only a few works report the depletion of GSH, which is antioxidant decreasing the free radical scavenging, increasing the therapeutic effects of CDT. Some of mitochondria-targeted therapies can be combined with innovative immunotherapy to achieve even better effects, mainly by achieving improved cytotoxicity with more TAAs released and resulting in a cascade amplification.

For various mitochondria-targeted nanomedicine enabled treatments, chemotherapy is proven to be a very effective treatment. However, the systematic toxicity to the entire body is significant. In contrast, PDT can effectively reduce toxicity but cannot achieve deep tissue penetration. SDT can achieve deep tissue penetration, but mechanical waves are less energetic compared to electromagnetic radiation, leading to low efficient ROS generation. RDT is also able to realize deep tissue penetration, yet the systematic toxicity to normal tissues cannot be ignored. Therefore, in a comprehensive view, PDT assisted with NIR light seems to be an optimized option, considering future clinic applications. To combat the limitation of insufficient penetration of NIR light, for those deep-seated tumors, phototherapy can be performed on deep tumor tissues with the help of tools such as fiber optics and endoscopes, in a noninvasive manner (Li C. et al., 2020). However, these technologies are still immature, so there is an urgent need to develop mitochondria-targeted photosensitizers and photothermal reagents suitable for *in vivo* application for clinical use.

In the future, firstly, except for PTX, newer physiologically active compounds and novel drugs that target mitochondria themselves are needed to be discovered and identified, which would make this concept straightforward and easier. Secondly, more work could be done in evaluating their realistic effects on tumor tissues *in vivo* to promote their potential clinical transitions. Thirdly, nanomedicine concurrently targeting of multiple intracellular compartments may be a direction to study the synergistic effects and their influences on amplifying therapeutic efficacy. Fourth, since hypoxia in tumor site microenvironment is an important factor attenuating the therapeutic efficacy, components able to be incorporated in nanoplatforms increasing the oxygen content or inhibiting the oxygen consumption that would boost the ROS generation to further damage mitochondria are desired. At the same time, smart constructions of nanomedicine are also needed to make clinical translation easier. This could be realized by taking advantages of superior materials integrating various functions. Fifth, the applications of mitochondria-targeted nanocarriers could also be expanded, for example, enhancing transfection efficiencies in gene delivery or gene therapy. Sixth, one of the major obstacles of mitochondria-targeted nanomedicine is short- and long-term cytotoxicity. The mechanism by which most groups can target mitochondria lies in their lipophilicity and positive charge, which has been studied. These positive charges affect mitochondrial membrane potential, interfere with the function of mitochondria, and inhibit ATP generation, thus further result in the cytotoxicity. Considering the above-mentioned reasons, mitochondria-targeted nanomedicine needs to be simultaneously targeted to cancer cells with high specificity, and reducing the damages to healthy tissues. There are mainly two means of highly and accurately targeting treatments to tumors: *1*) passively targeting effect (EPR) due to their nanosize, and the modification of the tumor-targeting ligand to actively accumulate in tumor tissues; *2*) direct intra-tumor injection in localized areas to carry out local therapy. Last but not least, mitochondria may influence the macrophage polarization by regulating intracellular ROS levels to participate in immunotherapy. Nowadays nanomedicine-based cancer therapies are majorly by directly acting on cancer cells or tumor tissues. However, the drawbacks of this direct killing of cancer cells or tumor tissues lie in that the drug loading efficiency in nanoparticles is relatively low and the nanoparticles are unable to efficiently penetrate solid tumors, which limit their therapeutic efficacies. With the increasing research breakthroughs in immuno-oncology, the recent trends of nanomedicine involve modulating immune cells, including tumor associated macrophages (TAMs) and dendritic cells, to restore immune cycles to fight against cancer. For instance, regulating the TAMs from M2 (proinflammatory) to M1 (anti-inflammatory) state corresponds to turning an immune suppression state into an immune active state. While ROS are important factor that regulate the macrophage depolarization, and the level of ROS are closely related to mitochondria. Thus, mitochondria may assist in immunotherapy by controlling intracellular ROS of macrophage polarization.

## Abbreviations

ATP, adenosine triphosphate; CDT, chemo dynamic therapy; DLCs, delocalized lipophilic cations; DQA, dequalinium; EPR, enhanced permeation and retention; HSPs, heat shock proteins; IMM, inner mitochondrial membrane; MMP, mitochondrial membrane potential; MPP, mitochondria-penetrating peptides; MPTP, mitochondrial permeability transition pore; mtDNA, mitochondria DNA; NIR, near infrared; OMM, outer mitochondrial membrane; PDT, photodynamic therapy; PTT, photothermal therapy; PTX, Paclitaxel; RDT, radiodynamic therapy; ROS, reactive oxygen species; RT, radio therapy; SDT, sonodynamic therapy; TPP, triphenylphosphonium.
